# Selected bacterial recovery in Trinidadian children with chronic tonsillar disease

**DOI:** 10.1016/S1808-8694(15)30152-X

**Published:** 2015-10-18

**Authors:** Lexley Maureen Pinto Pereira, Solaiman Juman, Isaac Bekele, Nadira Seepersadsingh, Abiodun A Adesiyun

**Affiliations:** a Dr (Professor of Pharmacology); b Dr (Lecturer in Surgery); c Dr (Senior Lecturer, Biometrics and Head, Department of Food Production); d Miss (Research Fellow); e Dr (Professor of Veterinary Public Health and Director, School of Veterinary Medicine)

**Keywords:** Children, tonsil surface and core, α haemolytic Streptococcus, ß haemolytic Streptococcus, Streptococcus pyogenes

## Abstract

Pharyngotonsillitis in children is widely treated with antibiotics. **Aim**: To examine tonsil surface and core microflora following elective adenotonsillectomy in children. **Methods**: Tonsils of 102 Trinidadian children were prospectively examined for surface and core bacteriological culture and identification between 2005-2006. **Results**: Tonsils (360) yielded 800 isolates of Streptococcus spp. (51.3%), Staphylococcus spp. (42.3%) and Gram-negative genera (6.4%). Surface and core recovery of staphylococci and streptococci were similar (p>0.05). More (p<0.001) surfaces (82.2%) than cores (63.3%) grew Streptococcus spp.; α-haemolytic Streptococcus prevalence was higher (p<0.001) than ß-haemolytic Streptococcus on surfaces (74.4% vs. 18.6%) than cores (58.9% vs. 13.7%). Surfaces and cores were not concordant for streptococci (p<0.0004) and α-haemolytic Streptococcus (p<0.007). Surface and core ß-haemolytic Streptococcus yield was higher (p<0.05) in 6-16 than 1-5 year olds (31% and 23.8% vs 12.5% and 8%). S. pyogenes surface and core prevalence was (84.6% vs 70%) and (50.0% vs 25.0%) in older and younger children respectively. Klebsiella spp. (6.6 %, 2.2%), Proteus (4.4%, 4.4%) and Pseudomonas (4.4 %, 1.1%) grew on surfaces and cores respectively.

## INTRODUCTION

Upper respiratory tract infections (URTIs) are the most frequent cause for patient visits in general practice, and in children account for a substantial proportion of family doctor consultations^1^. Sore throat, a predominant symptom of pharyngotonsillitis is one of the most common complaints in paediatrics^2^, inviting considerable antibiotic prescriptions. In the United States 10% of all antibiotic prescriptions for paediatric consultations are given for pharyngotonsillitis^3^. Most episodes of pharygotonsillitis are caused by viruses, and Group A β-haemolytic Streptococcus (GABHS) or S pyogenes accounts for just about 15% of all cases^2^.

Pharyngotonsillitis, one of the most common childhood URTIs remains clinically indistinguishable whether it is of viral or bacterial etiology. Variations in tonsillar microbial flora may increase the risk of repeated attacks of tonsillitis and adenotonsillar hypertrophy, and why some children are more susceptible to infections of Waldeyer”s ring is unclear. Tonsillectomy, alone or with adenoidectomy in selected children for symptoms of recurrent infection or for hypertrophy and snoring is one of the most frequent paediatric surgical procedures. At least 34-80% of patients with sore throat are estimated to have a ‘possible” bacterial aetiology^4^, ^5^ and approximately 24-65%, are actually tested for ß-haemolytic Streptococcus (BHS) in the throat^1^, ^6^.

In a current hypothesis, acute pharyngotonsillitis is caused by adherent bacteria on the tonsillar epithelium which remaing present in surface secretions on the tonsillar crypts^7^.

Surface bacteria on the tonsils may not represent resident bacterial carriage within the tonsils. Treatment based on organisms identified from surface swabs of tonsils after recurrent tonsillitis may only address the surface bacteria allowing resident bacteria in tonsil core tissue to persist. Hypertrophied tonsils also harbor bacteria, predisposing to recurrent infection. In Trinidad and Tobago antibiotics are widely used to treat children”s URTIs, and though general practitioners preferentially prescribe them while recognize their overuse in these conditions^8^. Information on bacterial carriage in childrens” tonsils in the Caribbean is not available. This study investigated the prevalence of aerobic organisms on tonsil surface and core tissues in children following elective adenotonsillectomy for recurrent and/or persistent symptoms of infection or sleep apnoea.

## METHODS

### Population

Children and adolescents (≤16 years) advised to undergo tonsillectomy for repeated attacks of tonsillitis and/or for hypertrophy causing obstructive sleep apnoea were eligible. Only those children who had not received any antibacterial agent in the past four weeks prior to planned surgery were recruited. A peritonsillar abscess excluded subjects from the study. Patients scheduled for surgery were enrolled as they presented at the study centers for medical attention once they met the inclusion criteria. Tonsillectomy was an indication for surgery if : (1) a child had experienced at least 4 recurrent attacks of tonsillitis in the past 12 months; (2) an otorhinolaryngologist diagnosed snoring and/or obstructive sleep apnoea was caused by hypertrophied tonsils with hypertrophied adenoids, in which case, both tonsils and adenoids were removed.

### Procedure

This was a prospective tri-centered study at two public sector institutes, one in north and the other in south Trinidad, and a nursing home. The Ethics Committee of the Faculty of Medical Sciences, The University of the West Indies (N^o^. EC22: 31/01-03/04). Parents/guardians were explained the nature and purpose of the study and consented to submit the dissected tissues for bacteriological examination. Care-givers were interviewed on a pilot-tested instrument before surgery to determine patient demographics, contributory family history, frequency of attacks of tonsillitis and a history of antibacterial therapy for the immediate past attack. Antiseptic and/or bactericidal agents were not used during surgery. Dissected tonsils collected in sterile airtight appropriately labeled containers were stored at 40C and transported to the laboratory for processing either on the day of surgery or within 24 hours. When this was not possible tissues were transported within 7 days of surgery and the cold chain was maintained till processing was completed.

### Bacteriology

Sterile swabs were applied separately to the left and right surfaces of the tonsils and sub-cultured on blood agar, MacConkey agar and chocolate agar. Plates were incubated at 37°C in 5% CO_2_ for 24-48 hours, cultures were processed for aerobic organisms and microbes were identified using standard methodology^9^. To detect microflora in the tonsil core, the surface was initially decontaminated by searing with a red hot metal scalpel blade, and then gently sectioned with an incision to reach the core of the structure. Sterile swabs were applied to the inner exposed left and right surfaces of the tonsils and sub-cultured on the stated media mentioned above.

### Statistical analysis

A patient was considered positive for bacterial growth if isolates were recovered from the surfaces and/or cores of at least one of the right or left tonsils Children were grouped into two categories of 1-5 years and 6-16 years. Results of bacterial analysis are presented for Staphylococci spp., Streptococci spp. and Gram-negative isolates. Streptococci were classed as α-haemolytic Streptococcus (AHS) and β-haemolytic Streptococcus (BHS). Responses from all samples were considered for bacteriological and data analyses without distinguishing the cause for tonsillectomy, based on previous reports which did not find significant differences between patients with recurrent tonsillitis and apnoea^4^, ^10^, ^11^. Matched pair statistical tools compared surface and core responses, and Chi-square tests were used for independent samples.

## RESULTS

Tonsils were removed in 51% (52) of children for chronic infection and in 45.1% (46) for obstructive tonsillar hyperplasia causing snoring and/or sleep apnoea. By chance, boys and girls were equally represented (50.0% each). About half the numbers of children were either below 6 years (52.9%) or between 6-16 years (48.1%) (Table1). The majority of patients were of either African (43.1%) or East Indian (32.4%) lineage. Fifty-five children (53.9%) had suffered at least one attack of sore throat in the past 12 months and of these 45 (81.8%) had suffered between 4 and 16 episodes. Forty-seven (52.2%) patients, (38 with recurrent tonsillitis and 9 with hypertrophy) had taken antibacterial agents for the immediate previous attack of sore throat. Amoxicillin alone or with clavulanate (40.4%) was most frequently prescribed followed by a macrolide (21.3%) {azithromycin (7, 70.0%), clarithromycin (2, 20.0%) and erythromycin (1, 10.0%)}. First generation cephalosporins were preferentially prescribed in 6 (12.8%) patients and one patient received cefuroxime. Only three respondents received prescriptions for penicillin V.

**Table 1:** Children”s (102) characteristics and features of sore throat.

Characteristic	Number (%) of patients
**Gender**	
Male	51 (50.0)
Female	51 (50.0)
**Age**	
1 - 5 years	54 (52.9)
6 - 16 years	48 (47.1)
**Ethnicity**	
African	44 (43.1)
Indian	33 (32.4)
Others^a^	24(23.5)
**Number of attacks of tonsillitis in the past 12 months**^c^	
None	20(19.6)
1-3	10(9.8)
≥ 4	45(44.1)
**Reasons for tonsillectomy**^d^	
Recurrent tonsillitis	52 (51.0)
Snoring	46 (45.1)
**Antibiotics taken for the last attack (47)**	
Amoxicillin/Co-amoxiclav	19(40.4)
Macrolides e	10(21.3)
Cephalosporins f	6(12.8)
Pen-V	3(6.4)
Others / No recall	9(19.1)

Information not available for 20 patients

a Includes one Caucasian, 3 Chinese and those with undeclared or unknown ethnicity

c Not known for 27 patients

d o information for 3 patients

e ncludes azithromycin, clarithromycin and erythromycin

f ncludes those who cefeclor, cefprozil and cefuroxime

### Overall frequency of isolates

Bacteriological data is available from 90 children, as some tissues (12 children) were discarded due to poor quality. Two (1.1%) of the 180 tonsils (90 left and 90 right tissues) were negative for aerobic bacteria. The overall frequencies of detection of Streptococcus spp. Staphylococcus spp. and other bacterial pathogens from surface and core tissues are seen in Table 2. Children grew more than one species of Streptococcus spp. and one isolate each of S. pneumoniae was recovered from the surfaces and cores. Overall the isolate yield was proportionally higher from surfaces (271) than from the cores (225).

**Table 2:** Overall frequency of isolation of bacteria from children”s tonsils ^a,b^.

Source of sample	Number of samples tested	Number of samples positive for:		
			Staphylococcus spp. d	Other bacteria^e^
Tonsil surface	180	126 (70.0)	116 (64.4)	29(16.1)
Tonsil core	180	90 (50.0)	115 (63.8)	20 (11.1)

Total	360	216 (60.0)	231(64.2)	49 (13.6)

Surface and core samples obtained from the left and right tonsils of 90 patients

Includes Streptococcus α-hemolyticus and β-hemolyticus and one each of S. pneumoniae on surfaces and cores

Tissues yielded multiple bacteria

d Includes 2 isolates of coagulase-negative staphylococci

e Includes 13 genera of Gram-negative bacteria

The 360 surface and core tissue samples yielded an overall of 800 aerobic isolates averaging 8.9 isolates per patient. Of these 800 isolates, 410 (51.3%), 338 (42.3%) and 52 (6.4%) were respectively positive for Streptococci spp., Staphylococci spp. and other bacteria. Streptococcal recovery of isolates (average 4.56) was numerically higher 70.0% (126) from surfaces, than from cores 50.0% (90). Surface (116, 64.4%) and core (115, 63.8%) tissue detection rates were similar for staphylococcal isolates. Fifty-two (6.4%) isolates of Gram-negative bacteria from 13 genera were recovered from the surfaces (16.1%) and cores (11.1%). The proportion of positive responses from the surfaces of children”s tonsils (Table 3) differed from the core responses for Streptococcus spp. (p<0.0004) and for AHS (p<0.007), but not for the BHS at the 5% level. Surface and core tissue growth of Staphylococci spp. was similar.

**Table 3:** Core and tissue responses of children (%) in whom cultures were not in concordance.

Surface	Core	Streptococcus spp	Streptococcus spp,		Staphylococcus spp,
			α	β	
Positive	Negative	22,2	22,2	7,6	5,6
Negative	Positive	3,3	6,6	2,3	6,7
p-value	<0,0004	<0,007	>0,093	>0,75	

### Recovery of Streptococci

More (p<0.001) tonsils were positive for Streptococcus spp. on the surfaces (82.2%) than the cores (63.3%) (Table 4). Streptococcal surface yield (74.4%) for AHS was higher (p<0.001) than that from the cores (58.9%). Similarly the surface yield for BHS (18.6%) was higher than the core yield (13.7%) at 10%. More patients (p<0.001) grew AHS compared with BHS on both surfaces (74.4% vs 18.6%) and cores (58.9% vs 13.7%). Only 2 isolates of S. pneumoniae, an unimportant aetiological agent in sore throat and pharyngotonsillitis, were recovered from surfaces.

**Table 4:** Frequency of recovery of streptococci and staphylococci from surfaces and cores of children”s tonsils.

Tissue source	Number (%) of children (n=90)					
	Staphylococcus spp.	Streptococcus spp.	p-value	Streptococcus spp.		
				A	B	p-value
Surface	62 (68,9)	74 (82,2)	< 0,06	67 (74,4)	19 (18,6)^a^	< 0,001
Core	64 (71,1)	57 (63,3)	> 0,5	53 (58,9)	14 (13,7)^b^	< 0,001
p-value	> 0,53	< 0,001		< 0,001	< 0,1	

a In the 19 patients Group A was present in 8 (42.1%), Group B in 3 (15.8%), Group C in (15.8%) and Group G in 8 (42.1%)

b In the 14 patients Group A was present in 8 (57.1%), Group B in 4 (28.6%) Group C in 2 (14.3%) and Group G in 6 (42.9%)

Group A and Group G streptococci were most frequently isolated from tonsil surfaces and cores. In the 19 children growing BHS on the surfaces, 42.1% (8) carried Group A and Group G each, and 15.8% (3) carried Group B and C each. In the 14 children with BHS recovery from the tonsil cores, 57.1% (8) grew Group A, 28.6% (4) grew Group B, 14.3% (2) carried Group C and 42.9% (6) yielded Group G.

### Recovery of Staphylococci

Surface (68.9%) and core (71.1%) staphylococcal recovery was similar (p>0.53) in children (Table 4). Though the number of children yielding staphylococci (71.1%) and streptococci (63.3%) from the cores was not significantly different, more children grew streptococci (82.2%) than staphylococci (68.9%) on the tonsil surfaces (p<0.06). Two isolates of coagulase-negative staphylococci were recovered, one each from a surface and a core of two children.

Age and recovery of Streptococcus spp. and Staphylococcus spp.

In children aged 1-5 and 6-12 years the respective staphylococcal surface and core recovery was 62.5% and 76.2% (Table 5). The overall frequency of recovery of Staphylococcus spp and Streptococcus spp. was not significantly (p > 0.05) affected by children”s age or the tissue site. However more (p<0.05) older children (6-16 years) harbored BHS on the tonsil surfaces (31.0% versus 12.5%) and the cores (23.8% versus 8.3%), compared with the younger age group (1-5 years). Irrespective of age (Table 5) carriage was higher for AHS than BHS on the surfaces (p<0.001) as well as the cores (p<0.001). More of older children (6-16 years) grew BHS on surfaces than cores (p<0.05). There was no yield of Group B BHS from either surface or core tissue in younger children, whereas, in older children (6-16 years) respective surface and core recovery was 23.0% and 40.0%. Irrespective of age, surface yield of GABHS was proportionately higher that that from the cores of younger (50.0% versus 25.0%) as well as older children (84.6% versus 70.0%). Although Group C BHS was recovered from the tonsil surfaces of more older (23.0%) than younger (16.7%) children, core tissue growth was recovered only from younger children (25.0%). Group G BHS displayed a similar pattern for surface growth with higher yield in older (46.1%) than younger (33.1%) children. Core recovery however was higher from younger (50.0%) than older (40%) children.

**Table 5:** Association between isolation of Staphylococcus spp. and Streptococcus spp. from tonsillar surfaces and cores of children selected for adenotonsillectomy with age.

Age group	Tissue source	Number (%) of children with tonsil samples positive for				
		Staphylococcus spp.	Streptococcus spp.	Streptococcus spp.		
					β	p-value
1-5 years	Surface	30 (62,5)	38 (79,2)	37 (77,1)	6 (12,5)^a^	< 0,001
(n=48)	Core	33 (76,2)	32 (66,7)	31 (64,6)	4(8,3)^c^	< 0,001
6-16 years	Surface	32 (66,7)	36 (85,7)	30 (71,4)	13(31,0)^b^*	< 0,001
(n=42)	Core	31 (64,6)	25 (59,5)	22 (52,4)	10 (23,8)^d^*	< 0,001

* p<0.05

a On the tonsil surface in 1-5 year olds (6 patients), Group A was present in 3 (50%), Group C in 1 (16.7%) and had Group G in 2 (33.3%)

b On the tonsil surface in the 6-16 year olds (13 patients) Group A was present in 11 (84.6%), Group B in 3 (23.0%), Group C in 3 (23.0%) and Group G in 6 (46.1%).

c On the core in the 1-5 year olds (4 patients) Group A was present in 1 (25.0%), Group C in 1 (25.0%), Group G in 2 (50.0%).

d On the core in the 6-16 year olds (10 patients) Group A was present in 7 (70.0%), Group B in 4 (40.0%) and Group G in 4 (40.0%).

### Recovery of other pathogens

Thirteen Gram-negative genera were isolated (Table 6). Proteus, Klebsiella, Chromobacterium Pseudomonas spp. and Alcaligenes spp. were recovered from either surfaces or cores (1% to 6%) of tonsils. Collective growth prevalence of the remainder microbes comprised Serratia, Acinetobacter, Neisseria spp., Enterobacter aerogenes, Enterobacter cloacae, Citrobacter and Flavobacterium from surfaces (5.0%) and cores (7.0%) respectively.

**Table 6:** Isolation of bacteria other than streptococci and staphylococci in children selected for adenotonsillectomy.

Tissue source	Number (%) of children (n=90) with tonsils positive for species of:					
	Proteus	Klebsiella	Pseudomonas	Chromobacterium	Alcaligenes	Others^a^
Tonsil surface	4 (4,4)	6 (6,6)	4 (4,4)	3 (3,9)	2 (2,2)	5 (5,5)
Tonsil core	4 (4,4)	2 (2,2)	1 (1,1)	1 (1,1)	1 (1,1)	7 (7,7)

a Includes isolates of species of Serratia, Haemophilus influenzae, Acinetobacter, Neisseria and Citrobacter, Flavobacterium Enterobacter aerogenes and Enterobacter cloacae

## DISCUSSION

We have found a high association of surface and core growth of AHS in adenotonsillectomized children with significant BHS carriage on tonsil surfaces and cores in older children. Approximately half the children studied were between 1-5 (53.3%) or (6-16) years (46.7%). Age is a predictor of the infective etiology of pediatric tonsillitis; viral infections are more common in children <3 years, and GABHS infections are more frequent in children ≥ 6 years^12^ Children most frequently received amoxicillin/co-amoxicillin and macrolides. In reports from Trinidad^8^ and Spain^13^, ^14^ amoxicillin-clavulanate and amoxicillin were most frequently prescribed for URTIs in primary care and penicillin V was never recommended even though it is the drug of choice to treat GABHS^3^. Steinman et al^15^ reported increased use of broad-spectrum antibiotics, especially azithromycin, clarithromycin and amoxicillin-clavulanate, in American children in primary care between 1991-99. The expanding empirical use of these agents for URTIs which have predominant viral aetiology indicates their unnecessary utilization.

Streptococcus spp. and Staphylococcus spp. were the most prevalent aerobes on children”s tonsils regardless of the site. Overall, children harbored similar proportions of aerobic bacteria on surfaces and cores with a total average of 8.9 isolates per child and respective surface and core averages of 3.0 and 2.5 isolates. Our findings compare with British findings^16^ of 9.2 and 8.8 average surface and core isolates respectively. However the sample was not limited to children. Italian children with recurrent tonsillitis who had not received antibiotics in the 20 days before sample collection, demonstrated a similar pattern of recovery for aerobic bacteria from tonsil surfaces (69.2%) and cores (59.2%)^17^

Overall streptococcal growth, and that of AHS in comparison to BHS was higher on the surfaces compared with cores. The high prevalence of surface and core AHS compared with BHS in oral and nasopharyngeal flora is expected. Brook et al^18^ reported Staphylococcus aureus, AHS and BHS were the predominant Gram-positive aerobic isolates on tonsil surfaces and cores in children with recurrent tonsillitis. Surow et al^19^ reported surface cultures grew normal respiratory flora but Staphylococcus was most commonly isolated from the core. High oral and nasopharyngeal AHS inhibits the growth of BHS in tonsillitis, and offers a mechanism to lower recurrence rates of streptococcal throat infections^20^, which are altered from antibiotic induced disturbances. The BHS carriage was consistent with reported peak age incidence of GABHS infections above 6 years^3^. Children are the major reservoir of GABHS and the target population for GABHS-induced pharyngotonsillitis and its suppurative or non-suppurative complications^21^ GABHS infections are common in school-aged children who mix with others in school and the neighbourhood^22^ forming a pool of high colonizers who can infect their peers. The lower frequency of BHS in younger children can be explained by their restricted peer contact as well as the residual presence of maternal antibodies.

Streptococcus pyogenes owes its major success as a pathogen to its ability to colonize, multiply rapidly and spread in the host while evading phagocytosis. We found considerably higher recovery of GABHS on tonsil surfaces (42.1%) and cores (57.1 %) than reported rates of 15.5%^23^, 21%^24^, and 28%^25^ from throats of apparently healthy children or those with recurrent tonsillitis (16.9%)^26^, ^27^ or tonsillar hypertrophy (20%)^21^, ^23^. Surow et al^19^ reported a 19.6% colonization rate in the tonsil core and Kielmovitch et al^26^ found high prevalence of S. pyogenes in cultured tonsil surfaces of children with obstructive tonsillar hypertrophy. The observed high rate of S. pyogenes colonization may have occurred because we excluded children who had received antibacterial treatment in the preceding 4 weeks, contrasting with Surow et al^19^ who limited this time to just one week before the study. S. pyogenes produces multiple adhesins with varied specificities which enable its ability to colonize niches in the upper respiratory tract^27^. The nature of these adhesins may differ in surface and core tissues in children, which can explain the non-concordance between surface and core growth of Streptococcus spp. and AHS. Researching the protein nature of these adhesins and their anchorage to the cell surface may elucidate the high rate of observed GABHS colonization in children. Nonetheless, our data suggest that Trinidadian children are high colonizers of S. pyogenes, and constitute a reservoir of GABHS providing a source of latent re-infection.

The most frequent non-GABHS isolates detected were Groups C and G, which are closely related, share virulence factors with GABHS and sporadically occur in acute pharyngotonsillitis as unusual pathogens^28^. They are generating interest as emerging nosocomial and opportunistic pathogens primarily for eye, ear or throat infections, cellulitis and rarely meningitis, septicemia, endocarditis, or glomerulonephritis^29^. Indian^25^ and Italian^30^ school children harboring BHS had high prevalence rates of 43.2% and 38.8% of Lancefield”s Group G BHS respectively. These findings in Caribbean children invite further research to elucidate BHS carriage in healthy children and those with pharyngotonsillitis. Low recovery of S. pneumoniae is supported by reports which failed to recover S. pneumoniae in pharyngotonsillitis^31^, URTIs and sore throat^23^.

The few studies reporting staphylococcal recovery in tonsillar flora in children do not demonstrate differences between pathological and normal tonsils. S. aureus was the commonest bacterium (9.9%) cultured from tonsils of 1000 healthy Israeli children <2 years^32^. Staphylococcal prevalence was similar with or without tonsil pathology in Brazilian^33^ (27% versus 28%) and Dutch^4^ children (6% in both groups). Gaffney et al^34^ isolated S. aureus from the tonsil core in 29% of children between 8-14 years with recurrent tonsillitis. Our observed higher prevalence of S. aureus on tonsil surfaces (68.9%) and cores (71.1%) has likely occurred because we cultured swabs from surfaces and cores of dissected tonsils in lieu of pharyngeal swabs which are neither reliable nor valid^7^, ^35^. Although the current observed dissimilarity in the frequency of staphylococcal detection compared with other studies may represent a true difference, it is pertinent to mention that differences in detection methodology and specimen handling prior to processing cannot be ignored.

H. influenzae, an infrequent Gram-negative isolate, has been demonstrated in sore throat^21^, tonsil hypertrophy and recurrent tonsillitis^17^, ^23^. In Sweden where routine throat swab analyses do not include H. influenzae, Gunnarsson^23^ has discussed its aetiological implications in pharyngotonsillitis. Mandatory immunization of children with Haemophilus influenzae type B vaccine in Trinidad and Tobago over the last decade would explain its absence in the present sample. Studies on large defined populations can clarify the aetiological role of H. influenzae in childhood tonsillitis in Trinidadian children. The several Gram-negative genera recovered in the present study have not been reported as far as these authors are aware. The isolation of Klebsiella and Pseudomonas species though at a low frequency, is worrying. Antibiotic exposure may have disturbed throat flora and facilitated re-colonization with these species. The precise reason why children acquired these organisms is unclear and their clinical relevance remains to be determined. Though the study was designed to examine aerobic bacteria, anaerobes and atypical bacteria cannot be ignored and need to be investigated.

The recovery of mixed aerobic bacteria from tonsil surface and core cultures raises the possibility that core bacteria may represent the surface bacteria. Exploring the relationship between core and surface aerobic bacteria Brodsky et al^10^ reported similar findings, but cautioned that surface bacteria may not truly represent the core growth. Other reports^7^, ^19^ do not reflect core bacteria on the surface tissue. Recurrent bouts of tonsillitis, significant inflammation, and bacterial adherence to mucosal epithelium or infection within the crypts may explain our differing results.

## CONCLUSIONS

We found higher streptococcal growth on tonsil surfaces than cores, and higher AHS than BHS yield from surfaces compared with the cores, Recovery of GABHS was high from both, surface and core tissues, particularly in older children. Trinidadian children are high colonizers of S. pyogenes, and constitute a reservoir of GABHS for latent re-infection. Studies to probe the mechanisms and influences of multiple adhesions of S. pyogenes in Trinidadian children are suggested.
